# Architecture of clathrin-independent AP3:ARF1-coated carriers

**DOI:** 10.1126/sciadv.aed1529

**Published:** 2026-05-15

**Authors:** Jonathan G.G. Kaufman, Grigory Tagiltsev, Danièle S. Stalder, Rebecca J. Taylor, Ioana Sava, Hui Guo, Katarzyna A. Ciazynska, Nathan R. Zaccai, Sally R. Gray, Yvonne Vallis, Stefan Höning, Bernard T. Kelly, David C. Gershlick, John A.G. Briggs, David J. Owen

**Affiliations:** ^1^ Cambridge Institute for Medical Research, Cambridge Biomedical Campus, Cambridge CB2 0XY, UK.; ^2^ Department of Cell and Virus Structure, Max Planck Institute of Biochemistry, Martinsried 82512, Germany.; ^3^ MRC Laboratory of Molecular Biology, Cambridge Biomedical Campus, Cambridge CB2 0QH, UK.; ^4^ Institute of Science and Technology, Klosterneuburg 3400, Austria.; ^5^ Institute for Biochemistry, Medical Faculty, University of Cologne, Cologne, Germany.

## Abstract

The AP3 complex mediates cargo sorting and carrier assembly for the trafficking of transmembrane proteins from endosomes to lysosomes. AP3 is generally believed to localize to clathrin-free, ARF1-positive, elongated carriers in cells, but the architecture of AP3-based coats was unknown. Using in vitro reconstitution and cryo–electron tomography, we demonstrate that AP3:ARF1 spontaneously remodels membranes containing cargo and the phosphoinositide PI(3,5)P_2_ into tubular structures coated in spiraling rows of AP3 arches and ARF1 dimers. Targeted point mutations disrupting critical AP3:ARF1 and AP3:AP3 lattice interfaces disrupt AP3 recruitment, carrier formation, and lysosomal cargo trafficking in cells. We propose that AP3 generates tubular carriers on endosomes by organizing ARF1 dimers into elongated membrane-deforming arrays while simultaneously selecting cargo. By demonstrating that AP3:ARF1 can generate carriers without using a clathrin lattice, we explain the clathrin independence of AP3-mediated trafficking.

## Teaser

AP3 generates clathrin-free carriers on endosomes by organizing via ARF1 dimers into elongated membrane-deforming arrays.

## INTRODUCTION

**Fig. 1.**
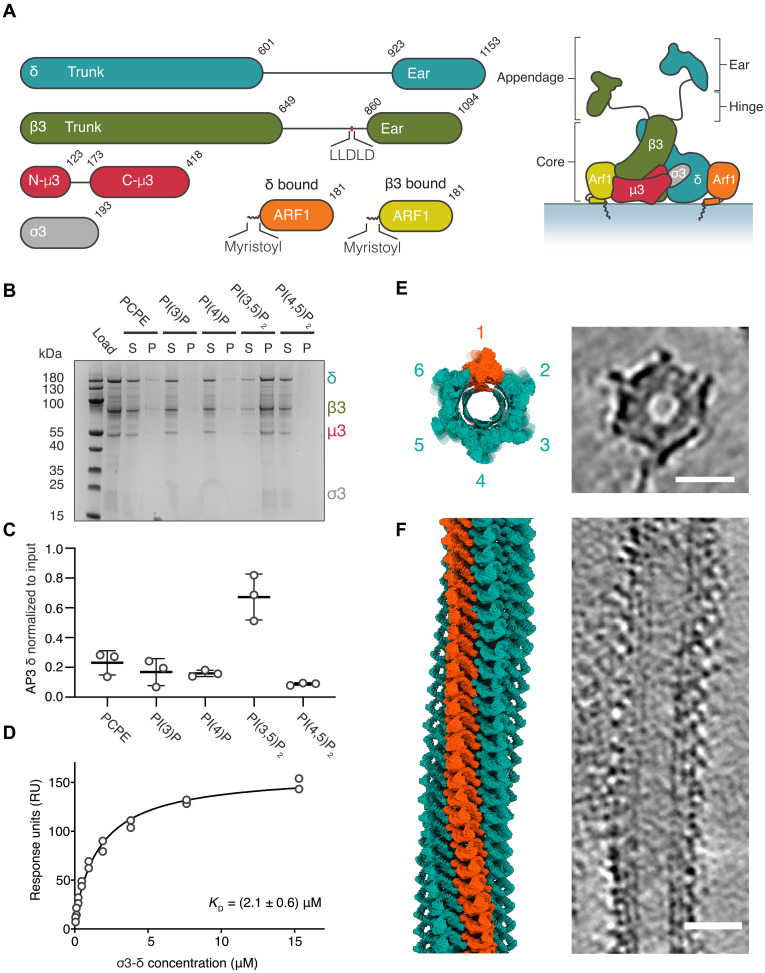
AP3 tubulates and coats PI(3,5)P_2_ and ARF1:GTP containing membranes. (**A**) Schematic diagram of the AP3 complex domain architecture and expected mechanism of membrane recruitment by ARF1. Subunits colored β3 (green), δ (blue), μ3 (red), σ3 (gray), β3-ARF1 (yellow), and δ-ARF1 (orange). (**B**) Liposome sedimentation assay of recombinant AP3 binding to liposomes enriched in the indicated phosphoinositides. Fractions labeled as (S) supernatant and (P) pellet. (**C**) Quantification of δ subunit band intensity in the pelleted fraction across indicated phoshoinositides from the liposome sedimentation assay, shown in (B). Liposome sedimentation assays were performed in triplicate (*n* = 3). (**D**) Liposome surface plasmon resonance (SPR) binding curve used to determine the affinity of fused σ3-δ hemicomplex to PI(3,5)P_2_ as 2.1 ± 0.6 μM. (**E** and **F**) Lattice map of a representative tubule viewed in cross section (E) or from the side (F). Produced by placing experimental densities for AP3 core and ARF1 at the positions and orientations determined by subtomogram averaging (STA). The pseudo-helical lattice has ~6.05 complexes per turn and a pitch of ~7.5 nm. AP3:ARF1 complexes (turquoise) with a single AP3 “stripe” highlighted (orange). Right hand panels show the equivalent slice through the cryo–electron tomogram. Scale bar, 20 nm.

In eukaryotes, the sorting and delivery of transmembrane and luminal cargo proteins to the correct organelles is mediated by coated carriers. A major class of cargo-selective coat proteins that mediate these processes are the heterotetrameric adaptor protein (AP) complex family, comprising AP1-5 adaptors, the coat protein complex 1 (COPI) F-subcomplex, and parts of TSET in plants (*1*, *2*). AP1-5 are composed of two large subunits (a β subunit and either α, γ, δ, ε or ζ subunit), a medium (μ) subunit, and a small (σ) subunit. The large subunits all consist of a stack of alpha solenoid domains, termed a trunk domain, followed by an appendage consisting of a ~100 residue flexible “hinge” and a C-terminal folded, “ear” domain (Fig. 1A). The σ and μ subunits both start with a longin domain, which in the μ subunit is followed by a short linker and a C-terminal Mu-homology domain (MHD), which itself is composed of two intercalated beta-sandwich subdomains, A and B (*3*). The β subunit trunk wraps around N-μ and the other large subunit around σ, together forming the adaptor “core.”

The best characterized AP family members are AP1 (β1, γ, μ1, and σ1 subunits) and AP2 (β2, α, μ2, and σ2 subunits). AP2 is central in clathrin-mediated endocytosis from the plasma membrane, whereas AP1 predominantly mediates retrograde transport from endosomes/post-Golgi tubular compartments back to the Golgi (*4*, *5*).

AP1 and AP2 both associate with polymeric clathrin through canonical clathrin box motifs (LΦxΦ[DE], where Φ is a bulky hydrophobic residue) positioned in the middle of their β-subunit hinges. AP1 and AP2 recruit transmembrane cargos containing YxxΦ motifs via their μ subunits and containing [ED]XXXL[LI] motifs via their σ subunits. While in the cytosol, AP1 and AP2 cores adopt a “closed” conformation in which their cargo binding sites are occluded by the β trunk, and in which the β hinge binds back to the core, rendering the clathrin box inaccessible (*6*).

AP1 is recruited to the membrane by binding the membrane-associated guanosine 5′-triphosphate (GTP)–bound conformation of the small guanosine triphosphatase ARF1 and by interacting with the phospholipid PI(4)P (*7*, *8*). AP2 recruitment is driven by binding membrane-associated FCHO1/2 and PI(4,5)P_2_ (*3*, *9*, *10*). PI(4,5)P_2_-binding sites are present in β2, α and on subdomains A and B of the μ2 MHD. For both AP1 and AP2, membrane attachment induces a large-scale relative motion of the large subunits and repositioning of the MHD. In the case of AP2, this relieves the autoinhibition of both the cargo and clathrin box binding sites and makes the cargo and PI(4,5)P_2_-binding sites coplanar. Thus, the conformational switch from a closed, cytosolic form to an open, membrane bound form couples AP membrane recruitment to cargo binding and coat polymerization.

Mammalian AP3 (subunits δ, β3, μ3, and σ3) (Fig. 1A) is recruited onto early/tubular endosomal compartments from where it sorts transmembrane protein cargo to endolysosomes, lysosomes, and a diverse group of cell type–specific compartments termed lysosome-related organelles (LROs) that include melanosomes and platelet-dense granules (*11*). The failure of AP3-dependent sorting leads to mislocalization of its cargoes. For example, tyrosinase fails to reach melanosomes, lysosomal associated membrane protein 1 (LAMP1) is rerouted via the cell surface and vesicle associated membrane protein 7 (VAMP7) relocalizes to the Golgi (*12*–*14*). Loss-of-function mutations in AP3 subunits cause a wide range of pathologies including Hermansky-Pudlak syndrome [pigmentation defects (albinism and blindness), pulmonary fibrosis, blood clotting defects, and immunodeficiency]. Loss of the brain-specific AP3 causes early onset epileptic encephalopathy and optic atrophy (*11*). Despite its importance, the mechanistic basis of AP3 function remains unclear.

Same as AP1 and COPI, AP3 is recruited to membranes primarily through interacting with ARF1: guanosine 5′-triphosphate (GTP). Within the cell, ARF1 is in excess over its AP family binding partners (*15*). Similarly to AP1 and AP2, AP3 recognizes cargoes containing YxxΦ and [ED]XXXL[LI] motifs, although it additionally recognizes VAMP7-containing SNARE complexes as cargo via its δ-hinge (*14*). However, studies on both yeast and mammalian AP3 suggest that, unlike AP1 and AP2, AP3 does not adopt an autoinhibited closed conformation in solution (*16*, *17*), and recent work on mammalian AP3 demonstrated almost no conformation differences between a solution structure and a structure bound to lipid nanodiscs via ARF1 in the presence of YxxΦ cargo (*16*). ARF1-mediated dimerization of the nanodisc-bound conformation to form β3-ARF1-ARF1-β3 was suggested to represent a possible scission intermediate (*16*).

Whereas AP1 and AP2 bind and polymerize clathrin triskelia to drive membrane vesiculation and hence carrier formation, it remains contentious whether AP3 uses clathrin or not. Despite AP3 having a clathrin box (*18*) in its β3 linker region, many studies demonstrate that AP3 only weakly colocalizes with clathrin on budding profiles (*12*, *19*–*21*). Furthermore, AP3 does not copurify with clathrin-coated vesicles (*22*), and experiments in cells indicate that the AP2 β2-ear, which is crucial for clathrin polymerization, cannot be functionally replaced with the β3-ear (*23*).

Thus, the machinery and mechanism by which AP3 can assemble coated carriers appears not to conform to the paradigms established by studies on AP1/clathrin and AP2/clathrin. Here, we set out to understand the mechanism by which AP3 and ARF1 assemble a coat to mediate trafficking.

## RESULTS

### Reconstitution of AP3 carrier formation in vitro

The binding of AP1 and AP2 to membranes is promoted by interactions with specific phosphoinositides, but the lipid-binding preference of AP3 has not, to our knowledge, been directly assayed. Using liposome-based sedimentation assays and surface plasmon resonance (SPR) with purified AP3 or with the purified AP3 subcomplex σ3-δ, we found a marked preference for binding liposomes containing PI(3,5)P_2_ over those containing PI(3)P, PI(4)P, or PI(4,5)P_2_ (Fig. 1, B to D, and fig. S1, A to C). Consequently, to reconstitute the formation of AP3-coated membranes in vitro, we mixed a purified, recombinant form of AP3 [full δ, μ3, and σ3; truncated β3 (residues 1 to 648)] with myristoylated-ARF1, the guanine-nucleotide exchange factor (GEF) ARNO, the nonhydrolyzable analog of GTP, GTPγS, and giant unilamellar vesicles (GUVs) containing PI(3,5)P_2_ and lipopeptides representing a YxxΦ motif-containing cargo (TGN38: CKVTRRPKASDYQRL) and a dileucine-motif containing cargo (MFSD12: SAGEHTPLLASSC), both coupled to lipids via maleimide chemistry (*24*). The reaction mixture was imaged by cryo–electron tomography (cryo-ET), revealing the spontaneous formation of ~40-nm-diameter protein-coated tubular structures scaffolding a ~20-nm membrane tube (fig. S1, D and E). Reconstructed tomograms revealed that cross section of these tubes is formed from six arch-shaped AP3 complexes forming one turn of a helix, (Fig. 1E) with the arches stacked “en face” along the length of the tube to generate long-pitch spiral stripes (orange in Fig. 1F).

**Fig. 2.**
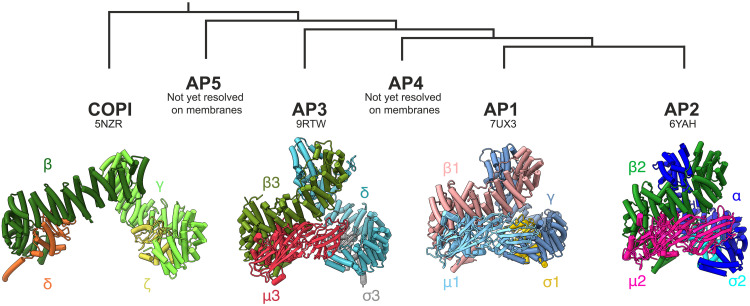
Comparison of core conformation for AP complex family resolved on lipid bilayers. Comparison of AP core conformation for AP family members resolved on lipid bilayers. Depicting range of extended open conformations from most extended to most compact. Left to right: COPI (5NZR), AP3 (this study), AP1 (7UX3), and AP2 (6YAH). Subunits colored as indicated.

To resolve the structure of AP3:ARF1 assemblies we performed reference-free subtomogram averaging (STA). Briefly, tubules were manually annotated, and initial positions were defined as a dense array on the tubule surface. From these positions, subtomograms were extracted and assigned an initial angle normal to the tubule surface. The subtomograms were iteratively aligned in SubTOM (https://github.com/DustinMorado/subTOM), and duplicates and poorly aligned subtomograms were removed. After 3-dimensional contrast transfer function (3DCTF) correction, subtomograms were refined in Relion 3.0 (*25*) before tilt-series refinement in M (*26*) and postprocessing in Relion 3.0 (see fig. S2 and Materials and Methods for further details). This resolved the structure of the assembled AP3:ARF1 tubular coat on membranes at 7.6 Å resolution (Fig. 2A and fig. S2). At this resolution, alpha helices are resolved, and the folded domains can be confidently identified and positioned within the structure. Within the coat, AP3 and ARF1 form an array of interactions with the membrane surface in addition to protein-protein packing interfaces that mediate helical lattice formation.

### AP3 conformation and membrane interactions in the assembled coat

In the assembled coat, AP3 adopts a wider, more extended “open” conformation than previously observed for AP1/2 on flat or tubular membranes (*27*, *28*) and much wider than observed for AP3 on lipid nanodiscs (*16*) but does not adopt the “hyper open” conformation reported for solution AP5 or membrane-bound COPI F-subcomplex (*29*, *30*) (Fig. 2). The known cargo binding sites on C-μ3 and σ3/δ are adjacent to the membrane, and the electron microscopy density suggests that they are occupied by cargo (fig. S3, A, B, E, and F). The subdomain A of C-μ3 contacts the membrane allowing for YxxФ binding; subdomain B points away from the membrane at ~20° (fig. S3H). This contrasts with previous observations of both C-μ1 (AP1) and C-μ2 (AP2), which lie completely flat on membranes, maximizing contact for lipid binding (*27*, *28*), and is consistent with subdomain B of C-μ3 lacking a number of the phosphoinositide-binding surface lysine residues that are present in C-μ2 (*31*, *32*).

In line with both Alphafold3 predictions and observations of AP3 on nanodiscs, we observe a density in the position of an amphipathic helix in the μ3_(139–148)_ linker that projects into the lipid bilayer (fig. S3G). This helix may aid both the membrane targeting of the AP3 coat and contribute to membrane deformation. The N-terminus of the δ subunit is positioned adjacent to the membrane (fig. S3, C and D)—the density observed linking δ to the membrane could represent PI(3,5)P_2_ (this position is the primary phosphoinositide binding site on other AP complexes), or it could be the additional amphipathic helix proposed by (*16*); however, the resolution of the density is insufficient to confirm this. Amphipathic helices have not been observed for other AP family members, so may be an AP3-specific feature, which could help to compensate for the absence of a membrane curvature-promoting outer scaffold layer (e.g., clathrin for AP1/AP2).

### Protein-protein interactions in the assembled AP3 coat

**Fig. 3.**
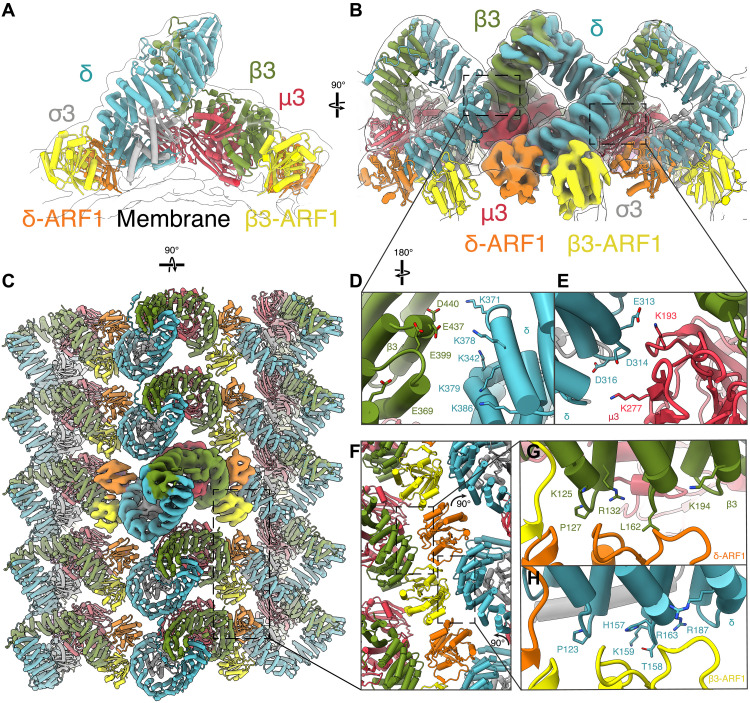
Architecture of AP3-coated tubules. (**A**) Transparent isosurface from a low-pass filtered (7.4 Å) reconstruction of AP3 and ARF1 assembled on membranes with the associated model docked and visualized in cartoon format. Colored as in Fig. 1A. (**B**) Rotated side view (180°) showing three consecutive AP3:ARF1 complexes within one stripe. The central AP3:ARF1 complex is shown as an isosurface of the 7.6-Å STA reconstruction, colored according to subunit. The same map is also shown as a lower-threshold translucent isosurface where the neighboring AP3:ARF1 complexes are visible. The neighboring AP3:ARF1 complexes are shown as molecular cartoons. (**C**) An expanded model to show the contacts within an AP3-coated tubule, viewed from the above the tubule. (**D**) Expanded panel of the left boxed region indicated in (B) showing the membrane-distal δ-β3 intercomplex contact. Residues are illustrated that form the acidic patch in β3 (green) and the basic lysine cluster in δ (blue). (**E**) Expanded panel of the right boxed region indicated in (B) showing the membrane proximal δ-μ3 intercomplex contact. Residues are illustrated that form acidic patches in δ (blue) and the basic lysine cluster in C-μ3 (red). (**F**) Expanded panel of the boxed region indicated in (C) showing the ARF1 dimer that forms the contacts between AP3 complexes in adjacent stripes. (**G** and **H**) Expanded panel of the boxed regions indicated in (F) comparing the residues forming the secondary ARF1-binding site on both (G) β3 and (H) δ.

The AP3:ARF1 lattice has a 1:2 stoichiometry. The six AP3 complexes in one helical turn around the tubule are interspersed with six ARF1 dimers. Their packing is such that stripes of AP3 and ARF1 dimers spiral around the long axis of the tube (Figs. 1, E and F, and 3, B and C). The interactions between adjacent AP3 stripes are mediated via ARF1 dimers, with no direct contact between AP3 complexes. The stacking of AP3 arches to create a stripe is mediated by two electrostatic interactions between AP3 and the neighboring AP3 complexes along the tube axis (n to “n + 6” and “n − 6” within the helical lattice). The first, a membrane distal interaction, occurs between a cluster of lysines on AP3 δ and complementary acidic residues on β3 (Fig. 3D). The second, a membrane-proximal interaction occurs between a small group of acidic residues on δ, which pack near a cluster of lysines on the raised subdomain B of C-μ3 (Fig. 3E). We suggest that this combination of two adjacent electrostatic patches contributes to determining the packing of AP3 on the membrane into its spiral lattice.

Within the assembled coat, ARF1 is bound via the switch1 and 2 regions to sites on AP3 β3 and δ, analogous to the binding of ARF1 to AP1 β1 and γ or the binding of ARF1 to COPI β and γ (fig. S4A). However, we observed additional interactions between a negatively charged patch on the opposite side of ARF1 and clusters of positively charged residues on β3 or δ (Fig. 3, F to H). The positively charged residues in β3 (K125/R132/R167/K194) and δ (H157/K159/R187/R163) occupy similar positions on either subunit. These residues are highly conserved among multicellular eukaryotes (*2*) and are conserved in both neuronal and non-neuronal β3 paralogues.

The net result of these interactions is to link adjacent AP3 complexes via two copies of ARF1 (Fig. 3C). The two copies of ARF1 form a C2-symmetric ARF1 dimer mediated by a short two-stranded β sheet created from portions of switch1 (Fig. 3F). This is similar to the dimerization interface observed for ARF1 on nanodiscs (*16*) and for ARF6 on tubulated membranes (*33*).

### ARF1 dimers mediate heterogenous linkage types between AP3 stripes

The AP3:ARF1 complex is ~twofold symmetric (δ-ARF1 is structurally homologous to β3-ARF1, such that when AP3 is rotated through 180°, the ARF1 molecules are in similar positions). Because the interactions between stripes are mediated solely by ARF1 dimerization, it would therefore in principle be possible for neighboring stripes to run in the same direction (parallel) or in opposite directions (antiparallel). For parallel stripes, ARF1 dimers would link β3 to δ heterotypically as in Fig. 3C: For antiparallel stripes, ARF1 dimers would link δ to δ or β3 to β3 homotypically.

**Fig. 4.**
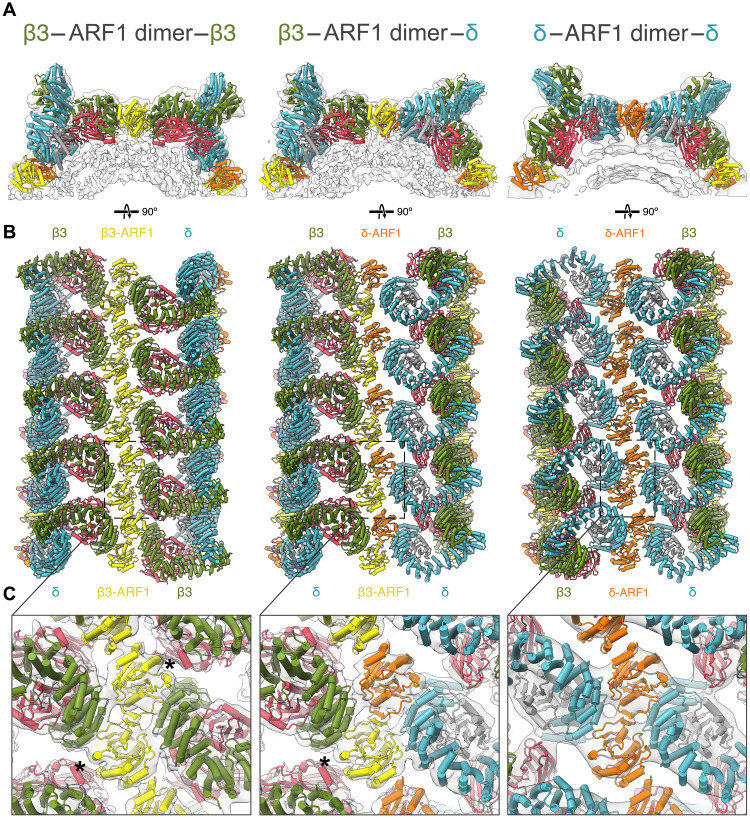
Heterogenous ARF1-dimer mediated linkages between AP3 complexes. (**A**) Transparent isosurfaces of reconstructions of AP3–ARF1 dimer–AP3 linkages viewed along the tube axis and fitted with models of the component proteins colored as in Fig. 2. Left: Homotypic β3–ARF1 dimer–β3 linkage. Center: Heterotypic β3–ARF1 dimer–δ linkage. Right: Homotypic δ–ARF1 dimer–δ. (**B**) Rotated view (90°) of the models in (A), viewed from outside the tube. (**C**) Expanded panel of the boxed regions illustrated in (B) illustrating the differences in the ARF1–C-μ3 contact (annotated by *).

The inspection of the positions of aligned subtomograms revealed a mixed population of parallel and antiparallel stripes, whereby both heterotypic and homotypic interactions were present to varying degrees within the same tubules. By analyzing the orientations of neighboring AP3 complexes, we resolved each individual AP3–ARF1 dimer–AP3 linkage (Fig. 4A). The homotypic β3–ARF1 dimer–β3 linkage gave the best resolved ARF1 dimer interaction, revealing the additional contact of the C-μ3 from adjacent stripes on either side of the ARF1 dimer (Fig. 4, B and C, fig. S5). This linkage is similar to that observed in a compact AP3 dimer formed on lipid nanodiscs, although the extended AP3 conformation observed in our data results in a different overall arrangement (Fig. 4B). While the heterotypic β3–ARF1 dimer–δ linkages only contribute a single C-μ3 to interact with the ARF1 dimer, the homotypic δ–ARF1 dimer–δ linkages lack this interaction entirely (Fig. 4C). We posit that the β3–ARF1 dimer–β3 interaction is the most stable, however it requires the production of an equal number of δ–ARF1 dimer–δ linkages between adjacent stripes to assembly a tubule (fig. S5). Therefore, it is likely that all contacts are tolerated and contribute to the formation of heterogeneously coated tubules. This ability to tolerate mixed linkage types reveals an unexpected flexibility in carrier formation, suggesting that a variety of productive carrier architectures may exist in cells.

**Fig. 5.**
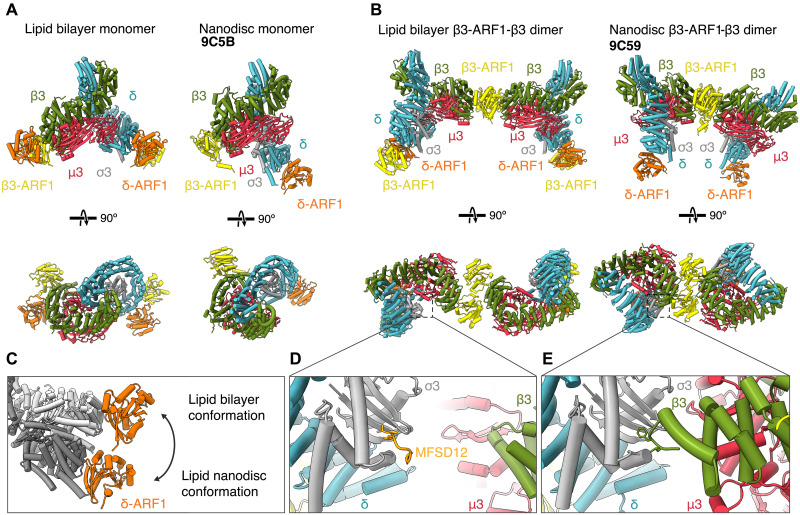
Structural comparison of AP3 conformation states on lipid bilayers and lipid nanodiscs. (**A**) Comparison of AP3 conformations resolved by STA on lipid bilayers and on lipid nanodiscs (9C5B) (*16*). Depicting difference in coordination angle of ARF1 coordination between S-shaped complex on lipid bilayers and C-shaped complex on nanodiscs. (**B**) Comparison of ARF1 dimer mediated β3–ARF1 dimer–β3 AP3 conformations between structure on lipid bilayers and nanodisc. Conformational difference results in vastly different radius of curvature around a membrane. (**C**) Conformational displacement of σ3δ hemicomplex between lipid bilayer (dark gray) and lipid nanodisc 9C5B (light gray), when structures are aligned to β3. Expanded panel from (B) showing the difference between presence and absence of dileucine cargo, (**D**) from STA showing the presence of dileucine cargo and absence of β3 N-terminus, and (**E**) from single particle of AP3 on nanodiscs showing the binding of pseudo-motif from β3 N-terminus binding across the dileucine binding site.

When assembled on lipid nanodiscs containing no cargo or only YxxΦ cargo, AP3 adopts a part open but still compact conformation similar to the conformation it adopts in solution (*16*, *17*). In this conformation, the dileucine binding site on σ3 is occluded by the N-terminal pseudo dileucine motif of β3, as has been similarly observed for AP1 and AP2 in closed conformations (*3*, *7*). In contrast, in our structure on tubular membranes, AP3 adopts a far more extended conformation, resulting in a rotation of the relative positions of the ARF1 binding sites with respect to each other (Fig. 5, A to C), and positioning the N-terminus of β3 too far from the dileucine binding site to forms a contact (Fig. 5, D and E). The formation of the extended conformation we observe is therefore likely driven by a combination of assembly on a bona fide lipid bilayer and by the presence of dileucine cargo displacing the β3 N-terminal dileucine pseudo-motif. Our structure suggests that the extended “active” conformation can subsequently polymerize to form carriers in the absence of clathrin.

### In cell validation of AP3 interactions

To establish the physiological relevance of the interfaces observed, we designed a set of mutations in δ and expressed them as fluorescent fusion proteins in the context of endogenous CRISPR–knockout (KO) of the δ gene, AP3D1. Immunoblots confirmed that all δ mutants were correctly assembled into a stable complex (fig. S6A). By fluorescence microscopy, δ as a fluorophore fusion localized to highly mobile, punctate structures throughout the cell (movie S1), consistent with previous findings.

**Fig. 6.**
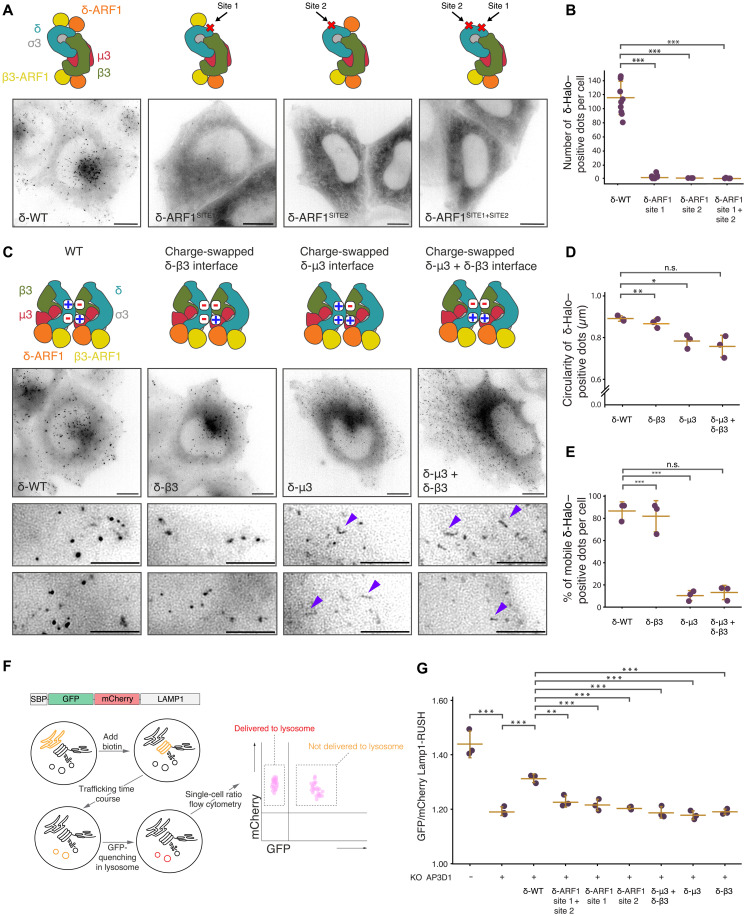
Effect of AP3D1 mutations on AP3 complex function. (**A**) Mutations in AP3D1 that abolish interaction with ARF1 sites relocalize δ-WT-SG to the cytosol. Lattice-SIM imaging of HeLa Cas9 cells expressing δ-WT-SG, δ-ARF1^Site1 + Site2^-SG (δF77S, δM110S, δL111S + δH157D, δK159D, δR187D, and δR163D), δ-ARF1^Site1^-SG (δF77S, δM110S, and δL111S) or δ-ARF1^Site2^-SG (δH157D, δK159D, δR187D, and δR163D). Endogenous δ was abolished using transient CRISPR-KO. Schematics indicate mutated ARF1-binding sites as red crosses. Scale bars, 10 μm. (**B**) Average number of δ-SG positive structures per cell. Three independent experiments were performed (10 to 15 cells quantified per condition). Tukey’s multiple comparisons test (post hoc, FWER = 0.05) was performed. ***P ≤ 0.001. (**C**) Schematics indicate charge pairs between neighboring AP3 complexes involved in stripe formation for WT AP3 and mutated δ. Mutations in δ that abolish interaction with AP3M1 and AP3B1 subunits affect endosomal/vesicular dynamics and shape. Lattice-SIM imaging of HeLa Cas9 cells expressing δ-WT-SG, δ-μ3 + δ-β3-SG (δE313K, δD314K, δD316K + δK342D, δK371D, δK378D, δK379D, and δK386D), δ-μ3-SG (δE313K, δD314K, and δD316K), or δ-β3-SG (δK342D, δK371D, δK378D, δK379D, and δK386D). Endogenous δ was abolished using transient CRISPR-KO. Scale bars, 10 and 5 μm in expanded insets. Blue arrows indicate tubular shaped δ-WT-SG structures. (**D** and **E**) Circularity (D) and mobility (E) of δ-WT-SG–positive dots determined from (C). Circularity was calculated with Amaris software. Mobility was established on ~100 puncate. Tukey’s test was performed. *P ≤ 0.05, **P ≤ 0.01, and ***P ≤ 0.001; n.s., not significant. (**F**) Schematic of Flow cytometry-based AP3 trafficking assay. (**G**) Flow cytometry analysis of GFP-mCh-LAMP1-RUSH trafficking to the lysosome. Endogenous δ was depleted in cell lines expressing δ-WT-Halo mutants. Lysosomal arrival is measured by quenching of the GFP signal after 1-hour biotin incubation (GFP/mCherry ratio). A minimum of ~1000 cells were analyzed per condition. Tukey’s test was performed. **P ≤ 0.01 and ***P ≤ 0.001.

A first set of mutations were designed to study the importance of AP3-ARF1 interactions. As expected, the mutagenesis of the established ARF1-binding site on δ (site1: δF77S + δM110S + δL111S) resulted in an entirely cytosolic δ subunit signal (Fig. 6A). The mutagenesis of the new ARF1 interface (site 2: δH157D + δK159D + δR187D + δR163D) also resulted in a cytosolic δ localization (Fig. 6A). These data indicate that δ requires both ARF1 interfaces for correct membrane recruitment (Fig. 6B).

A second set of three charge reversal mutations were designed in δ to study the interactions observed between neighboring AP3 complexes in the tubule without affecting AP3 membrane recruitment and assembly. AP3 δ-μ3 (δE313K + δD314K + δD316K) was designed to disrupt the δ-μ3 interface, AP3 δ-β3 (δK342D + δK371D + δK378D + δK379D + δK386D) was designed to disrupt the δ-β3 interface, and δ-μ3 + δ-β3 combines both sets of mutations. δ-β3 was recruited onto punctate structures similarly to δ–wild type (WT) (Fig. 6C). δ-μ3 and the double-mutant δ-μ3 + δ-β3 were also recruited onto membranes. However, the resulting carriers were more tubular in appearance and less motile than those observed following δ-WT complementation (Fig. 6, D and E). AP3 has been shown in other studies to localize to subdomains of ARF1-positive tubules (*20*). Thus, δ-μ3 and the δ-μ3 + δ-β3–elongated carriers may represent longer membrane tubules or alternatively may be due to the AP3 coating long sections of preexisting ARF1 tubules. This phenotype could therefore represent a failure of mutant AP3 to enrich into subdomains or a defect in tube scission. In either case taken together, these data indicate that the δ-μ3 lattice interface is essential for AP3 function.

To study quantitatively the functional output of the AP3 complex—delivery of membrane proteins to the lysosome—we have developed a quantitative flow cytometry–based kinetic delivery assay taking advantage of the RUSH (Retention Using Selective Hooks) system (*34*, *35*). Cells stably coexpressing streptavadin binding peptide-green fluorecent protein-mCherry-lysosomal associated membrane protein 1 (SBP-GFP-mCh-LAMP1) with streptavidin-KDEL were incubated with biotin for 1 hour, thereby freeing the SBP-tagged cargo from its streptavidin-mediated endoplasmic reticulum (ER) retention and initiating its transport through the secretory pathway. The depletion in the fluorescence of the LAMP1 GFP signal as it trafficked from the ER through the secretory pathway and endolysosomal system was then measured using flow cytometry, reflecting the quenching of SBP-GFP-mCh-LAMP1-RUSH upon reaching the acidic environment of the lysosome (Fig. 6F). As expected, the depletion of δ by CRISPR-KO significantly decreases the delivery of SBP-GFP-mCh-LAMP1-RUSH to the lysosome (Fig. 6G). The stable expression of a cDNA-encoding δ-Halo complementing the KO significantly recovers the phenotype (Fig. 6G). However, the exogenous expression of δ-Halo containing any of the mutations predicted to affect the interfaces between δ and ARF1, μ3, or β3 was unable to rescue SBP-GFP-mCh-LAMP1-RUSH trafficking to the lysosome (Fig. 6G), indicating the functional importance of the interaction interfaces for AP3 complex function.

## DISCUSSION

Previous studies assumed that AP3 would bind PI3P, which is enriched on early endocytic compartments that are also enriched for ARF1, and in the presence of cytosol, AP3 binds membranes containing PI3P or PI(3,5)P_2_ (*36*). We have identified a preferential, direct affinity of AP3 for PI(3,5)P_2_, which is characterized as being enriched on later endosomes/lysosomes (*37*). AP3 membrane recruitment using a combination of ARF1 (TGN/early endosome) and PI(3,5)P_2_ (late endosome/lysosome) conceptually parallels the recruitment of retromer, which is recruited by PI3P (early endosome) and RAB7 (late endosome/lysosome) (*38*, *39*). We speculate that by using coincidence detection of both earlier and later endosomal factors, AP3 recruitment can be constrained to a defined subdomain or subclass of maturing endosome.

We have demonstrated that AP3:ARF1 spontaneously forms coated tubules on cargo- and PI(3,5)P_2_-containing membranes and have resolved the architecture of the resulting helical AP3:ARF1 coat. The overall architecture of the resulting AP3:ARF1 lattice is reminiscent of the retromer coat, which also forms a tubular membrane coat from an array of alpha solenoid, arch-shaped scaffolds that link membrane-bending modules (*39*, *40*). The mutation of contacts critical for the AP3:ARF1 interactions identified in our cryo-ET structure led to a loss of AP3 recruitment to membranes. Mutations of contacts critical for the AP3:AP3 interactions identified in the structure result in the dysfunctional delivery of membrane proteins to the lysosome, implying the disruption of AP3-dependent trafficking. Despite being functionally defective, the δ-μ3 mutant carriers were morphologically similar to WT by fluorescence microscopy. The functional defect therefore presumably arises from a change in cargo selection or enrichment or vesicle formation defects below the resolution limit of the microscopy method. Abrogating the δ-μ3 contacts results in the formation of distended, nonmotile, tubular-coated membranes. We speculate these mutant structures are caused by a failure of AP3 to organize into functional, carrier-forming coats.

Although, by fluorescence microscopy, WT AP3 localization appears punctate, previous electron microscopy observations indicate the presence of elongated WT AP3 carriers in cells with diameters (~33 nm) similar to those formed in our in vitro system (*12*, *19*, *41*). Taking these observations together with our structural and mutagenesis data, we consider it likely that the in vitro AP3-mediated tubule formation recapitulates a structure involved in the formation of AP3 carriers in cells.

Thus far, all structures of membrane-bound AP family complexes interact with ARF1 via an evolutionary conserved primary site on either large subunit (COPI, AP1, and AP3) (*27*, *30*). In addition, all such complexes interact via a secondary site with additional copies of ARF1 from a neighboring complex to mediate the formation of a lattice (fig. S4). However, although this secondary site is evolutionarily conserved within a single AP complex, its location varies between members of the AP family (fig. S4). This points to distinct patterns of ARF1 coordination having evolved to tune carrier size and morphology and possibly to disfavor the assembly of mixed carriers, which would otherwise mislocalize cargos. We note that ARF3 has a negatively charged patch similar to the secondary-site binding patch on ARF1, suggesting that ARF3 may also be able to mediate AP3 oligomerization and consistent with observations that both ARF1 and ARF3 preferentially associate with AP3 (*42*, *43*).

The formation of the AP3:ARF1 coat arranges ARF1 dimers into stripes that run along the length of the coated tubules, resulting in a repeating, linear array of myristoylated amphipathic helices inserting into the membrane leaflet. These helices have been shown to promote membrane sculpting/curvature (*44*). Our AP3:ARF1 coat structure also suggests that there is an additional array of amphipathic helices is contributed by μ3 and possibly by δ. This corresponds to a total density of four amphipathic helices per ~166 nm^2^ of membrane surface. A combination of multiple amphipathic helix insertion, with possible additional contributions from scaffolding by the AP3:ARF1 lattice and molecular crowding effects between AP3 distal portions (*45*), is sufficient to induce membrane tubulation in our in vitro system. We suggest that these are also sufficient to induce formation of tubular membrane carriers in cell, without contributions from additional scaffolding proteins such as clathrin.

## METHODS

### Expression and protein purification of σ3-δ

Human σ3(_1–193)_-δ_(1–365)_-His6 was recombinantly expressed in BL21(DE3) pLysS *Escherichia coli* grown in 2xTY media, with expression induced with 0.2 mM IPTG at 22°C for 16 hours. Cells were resuspended in purification buffer [250 mM NaCl, 20 mM tris (pH 7.4), and 0.5 mM beta-mercaptoethanol (BME)] supplemented with AEBSF hydrochloride, MnCl_2_, and deoxyribonuclease I (DNase I). Cell suspensions were lysed using a cell disruptor (Constant Systems) at 30 kPa before clarification by ultracentrifugation (104,350 rcf) for 45 min. The resulting supernatant was batch bound to Ni–nitrilotriacetic acid sepharose (Cytiva) in purification buffer, the resin was washed with 400 ml of purification buffer, and the protein was eluted in purification buffer supplemented with 300 mM imidazole (pH 8). Eluted fractions were concentrated for gel filtration Superdex 200 HiLoad 26/60 column (Cytiva) into AP3 buffer [200 mM NaCl, 20 mM tris (pH 7.4), and 1 mM tris(2-carboxyethyl)phosphine (TCEP)]. Relevant fractions were pooled and concentrated for further use.

### Protein purification of Myr-ARF1

Myristoylated human Arf1 and human ARNO was prepared as described previously by (*46*, *47*).

### Insect cell cloning and expression

His_10_-AP3B1_(1–648)_ (*Pan troglodytes*) and AP3M1_(1–418)_ (*Rattus norvegicus*) were both cloned into pSPL vector, while AP3D1_(1–1203)_ (*Homo sapiens*) and AP3S1_(1–193)_ (*H. sapiens*) were cloned into the pFL vector. This was subsequently cloned into baculovirus following the Bac-to-Bac protocol (*48*). AP3B1_(1–648)_ lacks the appendage, reducing the sensitivity of the resulting protein complex to proteolysis during purification. This truncation does not affect membrane recruitment of AP3 or other AP complexes (*16*, *27*, *28*).

SF9 insect cells were cultured between 1 × 10^6^ and 3 × 10^6^ cells/ml in insect-Xpress protein-free insect cell medium (Lonza) at 27°C in shaking incubator. A 10 ml of P1 bacculovirus stock was added to 1 liter of SF9 cells at 3 × 10^6^ cells/ml, which were subsequently split to 1 × 10^6^ cells/ml. These cells were monitored for both yellow fluorescent protein signal and confluency until growth arrest was reached. Cells were harvested at 80% viability (3 days postarrest) by centrifugation 6240 rcf for 30 min before subsequently resuspended and stored at −80°C before use.

### Protein purification of AP3 complex

Insect cells pellets were resuspended in AP3 lysis buffer [200 mM NaCl, tris (pH 8.5), 10 mM imidazole, 1 mM MnCl_2_, 2 mM BME, DNase I (1 μg/ml), AEBSF (25 μg/ml) supplemented with PI Mix III, aprotinin, and soy trypsin inhibitor]. Cells were manually lysed by being sequentially passed through 19-, 21-, 23-, and 25-gauge needles (Agani needle). The resultant lysate was clarified by ultracentrifugation at (104,350 rcf) for 45 min, and the supernatant was collected. The supernatant was filtered using 0.2 μM Millipore filter (Merck). The filtered lysate was subsequently applied to a 1-ml HisTrap Excel column (Cytiva) connected using a S9H Sample pump (Cytiva) connected inline to an AKTA pure (Cytiva). After loading, the HisTrap was washed with 30 ml of AP3 purification buffer [200 mM NaCl, 20 mM tris (pH 8.5), 2 mM 2-mecaptoethanol, 10 mM imidazole, and AEBSF (25 μg/ml)]. The protein was eluted from the HisTrap in 0.5-ml fractions with AP3 elution buffer [200 mM NaCl, 20 mM tris (pH 8.5), 2 mM 2-mecaptoethanol, 300 mM imidazole, AEBSF (25 μg/ml)]. Relevant fractions were pooled and concentrated using a Vivaspin 20 100 kDa (Merck) for gel filtration. The protein was gel filtered into AP3 gel filtration buffer [200 mM NaCl, 200 mM tris (pH 8.0), and 0.5 mM TCEP]. Eluted peak fractions were analyzed by SDS–polyacrylamide gel electrophoresis (SDS-PAGE) and relevant fractions pooled if necessary.

### Lipid coupling of cargo peptides

Maleimide coupling of cargo peptides (MFSD12:GEHTPLLAPATC and TGN38: CKVTRRPKASDYQRL) was performed as in (*24*, *49*). In brief, a 1.5× molar excess of maleimidophenyl butyramide phosphoethanolamine (MPB-PE) 18:1 or MPB-PE 16:0 (avanti polar lipids) in chloroform was mixed 1:1 volume with a solution of cargo peptide (1 mg/ml) resuspended in 0.01 M Mops (pH 7.5). The coupling reaction took place under constant mixing of organic and inorganic phase overnight by end-over-end inversion. The reaction mixture was then evaporated under continuous nitrogen stream before resuspension in 2:1 chloroform/methanol. The yield of the reaction was assessed by intact-mass mass spectrometry.

### Liposome sedimentation assays

All lipid mixes subsequently were handled in a 95:5% chloroform/methanol solvent. A total of 1 mg of lipid mixes was made with either 80:20% phosphatidyl choline:phosphatidyl ethanolamine (PCPE) or 70:20:10% PCPE and a specific phosphoinositide species. Each lipid mix was subsequently dried down to a film using either argon or nitrogen streams under continuous rotation. To further ensure the removal of inorganic solvents, lipid films were held under constant vacuum for at least 2 hours before storage at −20 before use. Dried films were resuspended in 500 μl of AP3 buffer [200 mM NaCl, 20 mM tris (pH 7.4), and 1 mM TCEP) by being left to stand for 5 min and gently vortexed for ~5 s to create a multilamellar liposomes. A 8 μg of AP3 or 2 μg of the AP3 subcomplex σ3-δ was added to 50 μl of multilamellar liposomes suspension and incubated for at 4°C for 1 hour under gentle rotation. Protein-lipid mixes were spun down at 16,000 rcf for 10 min in a tabletop centrifuge before removal of the supernatant from the pellet. A 25 μl of (×4) Laemmli sample buffer was added to the pellet. A 20 μl of Laemmli sample buffer was added to 20 μl of supernatant. These were boiled at 95°C for 5 min before resolving by SDS-PAGE. SDS-PAGE was performed using the NuPage electrophoresis system (Invitrogen) and visualized using Instantblue Coomassie protein stain (ISB1L) (Merck). Sedimentation assays were performed in triplicate.

### Liposome SPR

A 1 mg of lipid mixes was made with either 80:20% PCPE or 76:20:4% PCPE, and a specific phosphoinositide species were generated. The lipid mix was subsequently dried down to a film under an argon stream. To further ensure the removal of inorganic solvents, lipid films were held under constant vacuum for at least 2 hours before storage at −20 before use. Lipid films were resuspended in 150 μl 0.3 M sucrose for 1 hour with intermittent gentle vortexing to produce sucrosomes. Sucrosomes were then diluted in 1 ml of deionized water before pelleting at 16,000 rcf for 30 min. The supernatant was removed, and the pellet was resuspended in SPR loading buffer [500 mM NaCl, tris (pH 8.7), and 1 mM TCEP] before extrusion using Avanti mini extruder (Avanti Polar lipids) through a 200-nm pore membrane (Wattman). Liposomes were applied to an L1 chip (Cytivia) in SPR loading buffer until a response of 1000 was obtained. The binding of σ3-δ to liposomes species was performed in AP3 buffer [200 mM NaCl, 20 mM tris (pH 7.4), and 1 mM TCEP].

### GUV electroformation

For GUV electroformation and subsequent AP3 reconstitution, a lipid mix of 51% 1-palmitoyl-2-oleoyl-sn-glycero-3-phosphocholinem (POPC), 26% DOPE, 7% DOPS, 10% cholesterol, 2% PI(3,5)P_2_, 1.5% MFSD12-lipidated, 1.5% lipidated-TGN38, and 1% DiI was made up in chloroform before being dried down under continuous nitrogen stream. The dry lipid film was dissolved in 25 μl of chloroform/methanol (2:1). A 10 μl of the lipid mix was spread across one indium tin oxide (ITO)–coated slide (Sigma-Aldrich). The lipid mix was dried under nitrogen stream. An electroformation chamber was created by placing a silicone gasket around the dried lipid mix and a second ITO-coated slide facing the gasket, the entire assembly was held together using a paper clamp. A 650 μl of 0.5 M sucrose was added to the chamber. Plates were warmed to average transition temperature of the lipids (~65°C) before beginning overnight electroformation (2.5-V amplitude and 10-Hz sinusoidal voltage). The following morning, the GUVs were detached from the ITO-coated slide and resuspended in 0.5 ml of 0.5 M glucose and allowed to settle overnight at 4°C.

### AP3 complex membrane reconstitution

For reconstitution of AP3 tubules, GUVs (200 μM lipid concentration) were incubated with 1.2 μM AP3, 6.2 μM myr-ARF1, 2 μM MgCl_2_, 2 μM GTPgS, and 3.6 μM ARNO in a 20*μl reaction and left to stand for 1 hour at room temperature. The supernatant, which contained AP3-coated tubes, was taken for cryo-EM sample preparation.

### Cryo-EM sample preparation

All grids were plunge frozen using a Vitrobot Mark IV, operated at 100% humidity and 18°C. C-flat CF2/2 Cu300 grids were glow-discharged at 25 mA for 60 s before use. A 4 μl of reconstituted AP3 tubules was applied to grids and blotted for 4 s with a blot force 3 before plunge freezing in liquid ethane/propane (1:1).

### Cryo-ET

Tilt series were collected on a Titan Krios G4 cryo-Electron TEM (TFS) operated at 300 keV equipped with a SelectrisX energy filter and Falcon4i direct electron detector. Tilt series were collected between −60° and +60° in 3° angular increment from 0° tilt angle using dose symmetric scheme in EPU Tomo5 (TFS). Images were taken with a resulting pixel size of 1.548 Å and a total accumulated dose of ~120 e^−^/Å^2^ with a target defocus range between −3.0 and −6.0 μm in 0.2-μm steps.

### Tomogram reconstruction and STA

Raw EER files of tilts were motion corrected and aligned into an initial tilt series in subtom_preprocess.sh [subTOM package (v1.1.5) (https://github.com/DustinMorado/subTOM)]. These tilt series were then aligned and reconstructed as bin4 tomograms using IMOD version (4.11.19).

A total of 528 AP3-coated tubes were manually picked from 32 tomograms in UCSF Chimera (*50*) with the tomograms binned to a pixel size of 6.191 Å (bin4). The tubular morphology and diameter were manually annotated using a chimera plugin (www.biochem.mpg.de/7940000/Pick-Particle) (*51*). Initial coordinates were seeded perpendicular to the surface of the annotated tubule with an even spacing of 7.43 nm (12 pixels in bin4 tomograms), resulting in eight subtomograms per turn. From 1 tomogram, bin4 subtomograms (48 pixel box size) were extracted and aligned with iterative reference free alignment using subTOM package (v1.1.5) (https://github.com/DustinMorado/subTOM) to obtain an initial reference volume. The resulting reference was used for reference-based alignment of the complete dataset but with addition of allowing a 180° in-plane rotation. Duplicates were removed, and poorly aligned positions were cleaned on the basis of the cross-correlation score and manually, resulting in a final set of 65,352 subtomograms. Aligned tilt series and subtomogram coordinates were imported to Warp (v1.0.9) (*52*) for CTF estimation and correction. Subtomograms and per-particle CTF models were extracted at bin1.55 (2.4 Å/pixels) and refined in RELION (v3.0) (*25*), yielding a 13.6-Å resolution map. Subtomograms with refined coordinates and angles were used to refine the tilt series alignment in M (v1.0.9) (*26*). New subtomograms were extracted at bin1.42 (2.2-Å pixel size), and particle positions were cleaned in using the aligned particle positions before local refinement yielding an 8.4-Å resolution structure. These particles were transferred back to M for another round of refinement of the tilt series alignment. The resulting halfmaps were postprocessed in RELION yielding a 7.6-Å resolution structure.

Using the aligned positions from the complete particle list, positions of neighboring particles were sorted according to their relative orientation as described in (*28*). From these positions the coordinates were rotated then shifted to centre on the ARF1:ARF1 interface for β3–ARF1 dimer–β3, δ–ARF1 dimer–β3 and δ–ARF1 dimer–δ using rotate_particles_star.py, starpy (https://github.com/fuzikt/starpy). In the case of β3–ARF1 dimer–β3 and δ–ARF1 dimer–δ, duplicate particles were removed, and in subsequent refinements, C2-symmetry was enforced. These positions were subsequently locally refined in RELION before tilt refinement in M. The resulting halfmaps were postprocessed in RELION yielding maps of β3–ARF1 dimer–β3 at 8.5-Å resolution, δ–ARF1 dimer–β3 at 8.9-Å resolution, and δ–ARF1 dimer–δ at 13-Å resolution.

### Model building

Alphafold 3 (AF3) (*53*), was used to generate five models of mammalian AP3 in complex with two copies of ARF1 in the GTP bound conformation. As the structure from STA was in a much more extended conformation than AF3 models or previous structures, the direct rigid body docking of the complete AF3 model into the STA density was not possible. To create the C1 model the predicted complex was separated into three submodels, each of which was independently fitted into the map as a rigid body. Submodel 1 consisted of σ3 (1 to 193), δ (1 to 387), and δ-ARF1 (1 to 181). Submodel 2 consisted of μ3 (1 to 418), β3 (1 to 472), and β3-ARF1. Submodel 3 consisted of β3 (473 to 636) and δ (388 to 638) formed submodel 3. Residues 606 to 638 from the δ-hinge were predicted by AF3 to bind across the vertex of the β3-solenoid. Our structure contained electron microscopy density corresponding well to the predicted positions of these residues, and they were therefore included in the molecular model. This assignment is further supported by the absence of this additional density in the published structure of AP3 lacking the δ-hinge (*16*, *17*). Rigid body fitting was performed using ChimeraX (*54*–*56*). This positioned truncated regions of submodels 1 and 2 in close proximity to their adjoining regions in submodel3; thus, the split β3 and δ chains were rejoined into single chains before real-space refinement in COOT to correct bond angles, distances, and torsions. Using the previously resolved ARF1 dimer (Protein Data Bank ID: 9C5A), ARF1 was first superimposed with the existing ARF1 on β3 and δ before rigid docking into the EM density. Flexible regions not accounted for sufficiently by modelable density were removed, these regions are listed as follows; β3(1 to 42), β3(259 to 292), δ(1 to 17), σ3(154 to 193), and ARF1(1 to 13). Side chains and rotamers were not modeled and were therefore removed in ChimeraX. For modeling of the ARF1-dimer–mediated linkages, two copies of the C1 model were fit as rigid bodies into the β3–ARF1 dimer–β3, β3–ARF1 dimer–δ, or δ–ARF1 dimer–δ linkage, and duplicated ARF1 dimers were removed.

### Antibodies and other reagents

The anti–AP-3δ (mouse-SA4, provided by A. Peden; reference no. 10.1083/jcb.200311064), the Rabbit monoclonal anti-AP3M1 antibody [EPR16385] from Abcam (ab201227) and AP3B1 (E7U2P) Rabbit monoclonal antibody from Cell Signaling Technology (#95765). The secondary antibodies used were donkey anti-mouse IgG Alexa 790 (ab175780) and donkey anti-rabbit Alexa 680 (ab175772) were from Abcam (Cambridge, UK). The following cell-permeable dyes were obtained from these vendors: 646 HALO Dye (GA112A; Promega).

The following antibiotics were used to select the newly generated stable cell lines: hygromycin B (250 mg/ml; 10687010; Invitrogen) and puromycin (1 μg/ml; A1113803; Gibco). The following chemicals were used in this work: biotin (B4501; Sigma-Aldrich).

### Plasmids

For transient KO cells, guide RNAs targeting AP3D1 (table S2) were cloned into pKLV-U6gRNA (Bbs I)-PGKpuro2ABFP (Addgene, plasmid #50946) using the Bbs I restriction sites. The CRISPOR guide RNA tool (*57*) (https://doi.org/10.1093/nar/gky354) was used to custom-design two guide sequences per gene of interest. The Cas9 viral expression backbone was a gift from P. Lehner and the packaging vectors pMD.G and pCMVR8.91.

To generate the different piggybac vectors, we first created a piggybac-CMV-StrepKDEL-IRES-SBP-Halo and a piggybac-CMV-StrepKDEL-IRES-SBP-GFP [described in (*35*)]. To generate the piggybac-CMV-StrepKDEL-IRES-SBP-GFP-mCh-LAMP1WT construct, the piggybac-CMV-StrepKDEL-IRES-SBP-GFP vector was opened with Xba I, and the PCR products of mCherry and LAMP1WT were Gibson assembled into it. We then generated a universal piggybac-CMV-Halo backbone by digesting the piggybac-CMV-StrepKDEL-IRES-SBP-Halo vector with Afe I and Bam HI and annealed two primers to reconstitute the two restriction sites. We also generated a universal piggybac-CMV-mStayGold-HA backbone by digesting the piggybac-CMV-Halo backbone with Afe I and Xba I to remove the Halo and the sequence of mStayGold (aka StayGold QC2–6 FIQ, which was codon optimized and synthesized), and the PCR product of HA was Gibson assembled into it. Next, guide-resistant AP3D1 was cloned in-frame upstream of Halo or StayGOLD-HA by using the Afe I site. The different AP3D1 mutants were cloned in a similar way except that a combination of PCR products and gblocks was used (Integrated DNA Technologies, containing the appropriate set of mutation combinations).

### Cell lines and lentiviral particles production

HeLa cells were already available in the laboratory, and Lenti-X 293T cells were obtained from Takara Bio (632180). Cell lines were grown in Dulbecco’s modified Eagle’s medium (DMEM) high glucose (D6429, Sigma-Aldrich) supplemented with 10% fetal bovine serum (F7424, Sigma-Aldrich) and MycoZap Plus-CL (VZA-2012, Lonza) and were kept at 37°C in a humidified 5% CO_2_ atmosphere. The stable HeLa cas9 cell line was generated as described in (*35*).

AP3D1-StayGold and mutant HeLa Cas9 cell lines used for imaging were generated as follows. HeLa Cas9 (0.4 × 10^6^) cells were seeded on a six-well dish. The next day, cells were transfected with transposase (pJEx21) together with piggybac-CMV-AP3D1-StayGOLD-HA WT or mutant (one-half in favor of the transposase). Two days later, positive cells were selected with hygromycin B and fluorescence-activated cell sorting–sorted ~2 weeks later for StayGOLD-positive cells.

The cell line used for the trafficking assay to the lysosome was generated as detailed above except that HeLa Cas9 cells were transfected with transposase together with piggybac-CMV-StrepKDEL-IRES-SBP-GFP-mCh-Lamp1WT. The resulting GFP-mCh-LAMP1WT-RUSH cell line was then transfected in a second step with transposase together with piggybac-CMV-AP3D1-Halo WT or the different mutants of AP3D1.

Lenti-X 293T cells were used to package the pKLV-puro vectors encoding plasmid/guide RNAs into lentiviral particles as previously described (*58*). Viral supernatants were harvested after 48 hours, filtered through a 0.45-μm filter, and when needed, concentrated down 10 times using the Lenti-X Concentrator (631232, Takara Bio). Supernatants were kept at −80°C before being directly applied to target cells, which were then spun at 700*g* for 1 hour at 37°C. When necessary, cells were transiently selected with the appropriate antibiotic 48 hours after transduction.

### Live cell imaging

Transient CRISPR KO was achieved by transducing previously established stable Cas9 cell lines. These include HeLa-Cas9 AP3D1-SG WT and mutants. Briefly, 25 × 10^3^ cells were transduced with 200 μl of lentiviral supernatant in a 48-well plate. Forty-eight hours posttransduction, cells were replated in a six-well plate and incubated with puromycin for an extra 4 days. On day 5, cells were detached with trypsin, and 9 × 10^4^ cells were plated onto Matrigel-coated glass coverslips (CB00250RAC, Menzel-Gläser). Two days later, cells were imaged in an Elyra7 with Lattice SIM^2^ microscope (Zeiss) equipped with an environmental chamber (temperature controlled at 37°C, humidified 5% CO_2_ atmosphere), two PCO.edge sCMOS version 4.2 (CL HS) cameras (PCO), solid state diode continuous wave lasers, and a Zeiss Plan-Apochromat 63×/1.4 Oil DIC M27 used for lattice-SIM, all under the control of ZEN black software (Zeiss).

Fiji software was used to generate time colour-coded maximum projections, create kymographs, and measure the mobility of AP3D1-positive dots. The circularity was calculated with the Bitplane Amaris software (10.1.0). One frame of each independent repeat was analyzed by using the surface tool to detect AP3D1-positive dots (surface detail was set at 0.08 μm), and the background was subtracted. The circularity of the dots was determined by the software on a minimum of 100 dots in each frame.

### Trafficking assay to the lysosome of GFP-mCh-LAMP1-RUSH

GFP-mCh-LAMP1WT-RUSH HeLa Cas9 cell line expressing the different AP3D1-Halo mutants were depleted of endogenous AP3D1 as described for live-cell imaging except no puromycin was applied on the cells on day 3. On day 6, cells were incubated in complete DMEM media containing 646 HALO Dye (20 nM; GA112A, Promega). On day 7, cells were detached with trypsin and resuspended in DMEM containing 25 mM Hepes (25 mM; 15630080, Gibco). Each well was split into two 1.5-ml microcentrifuge tubes and incubated at 37°C in a heating block (DB200/2, Techne). d-biotin (B4501, Sigma-Aldrich) at a final concentration of 500 μM was added for 1 hour in one tube of each condition. Cells were then incubated on ice for 5 min to stop RUSH and subsequently spun down (4°C, 500*g*, 5 min) to remove the supernatant. All downstream manipulations were either on ice or at 4°C. The cells were then washed once with 500 μl of phosphate-buffered saline and filtered using the Cell-Strainer capped tubes (352235, Falcon). A minimum of 1000 cells per sample was analyzed using an LSRFortessa cell analyzer (BD Biosciences), gating for mCherry-positive cells (indicative of GFP-mCh-LAMP1-RUSH expression) plus any other concomitant fluorophore when appropriate (646-positive cells are indicative of AP3D1-Halo expression and blue fluorescent protein (BFP)-positive cells of guide RNAs expression). Data were analyzed using FlowJo software (v10.8.1). RUSH was inferred by single-cell plotting the relative intensity of GFP (thus assaying the GFP quenching of SBP-GFP-mCh-LAMP1WT in the acidic environment of the lysosome) over that of mCherry (total SBP-GFP-mCh-LAMP1WT). To better reflect the extent of GFP quenching, the values were then transformed as follows: 1/(GFP:mCherry).

### Immunoblotting

HeLa Cas9 cell line expressing the different AP3D1-SG mutants were depleted of endogenous AP3D1 as described for live cell imaging. On day 7, cells were collected for Western blotting as previously described (*59*). Briefly, cells were lysed in NP40 buffer [50 mM Hepes (pH 7.4), 150 mM NaCl, 1% NP-40, and 1× cOmplete protease inhibitor cocktail (Roche Diagnostics)] before boiling in TruPAGE lithium dodecylsulphate (LDS) sample buffer (Merck) supplemented with 0.1 M dithiothreitol for 10 min. SDS-PAGE was performed on Millipore mPAGE 10% Bis-Tris Precast Gels (MP10W12) using the omniPAGE Mini Vertical Protein Electrophoresis System (Cleaver Scientific, Rugby, UK). Following the transfer of proteins using a Mini-PROTEAN 2-D Electrophoresis Cell (Bio-Rad, Hercules, CA) to Immobilon-P PVDF membranes (Merck-Millipore, Watford, UK) for fluorescence detection, membranes were blocked with 5% dried skimmed milk powder in tris-buffered saline with Tween 20 (TBS-T). Incubations with primary antibodies (1:500 dilution) were carried out overnight at 4°C, with Alexa-conjugated secondary antibodies (1:2000 dilution) applied subsequently for 1 hours at room temperature. The fluorescent detection of bound antibody was carried out by scanning with an Odyssey CLx (LI-COR Biosciences, Lincoln, NE), followed by immunoblot quantification using Image Studio Lite 5.2.5 (LI-COR).

### Statistical analysis (fluorescence microscopy)

Statistical analysis was performed using Python 3.7 or GraphPad 8 statistical significance was considered when *P* < 0.05. Comparisons were made using multiple comparison of means with Tukey post hoc, family-wise error rate (FWER) = 0.05. All quantitative data are expressed as means ± SD of at least three independent experiments.
