# Supplementary material for: Architecture of clathrin-independent AP3:ARF1-coated carriers
Source: Sci Adv. 2026 May 15;12(20):eaed1529. doi: 10.1126/sciadv.aed1529 (PMC13178560; doi:10.1126/sciadv.aed1529)
Supplement: Supplementary file 1 — Figs. S1 to S6 Tables S1 and S2 Legends for movies S1 and S2 [file sciadv.aed1529_sm.pdf]

Science Advances
AAAS

Supplementary Materials for

Architecture of clathrin-independent AP3:ARF1-coated carriers

Jonathan G.G. Kaufman *et al.*

Corresponding author: David C. Gershlick, dg553@cam.ac.uk; John A.G. Briggs, briggs@biochem.mpg.de; David J. Owen, djo30@cam.ac.uk

Sci. Adv. **12**, eaed1529 (2026)
DOI: 10.1126/sciadv.aed1529

The PDF file includes:

Figs. S1 to S6
Tables S1 and S2
Legends for movies S1 and S2

Other Supplementary Material for this manuscript includes the following:

Movies S1 and S2

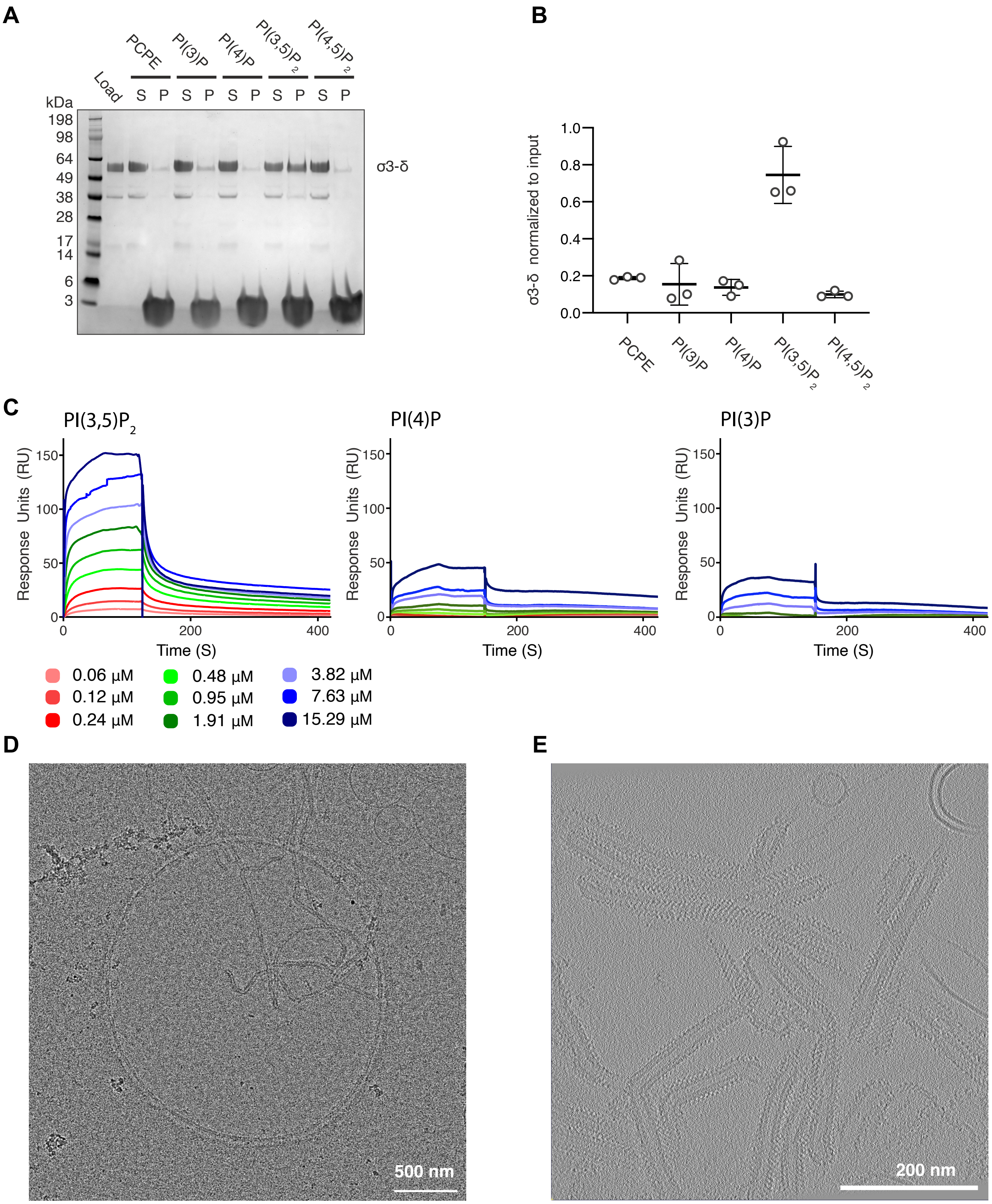

Fig. S1. Binding of AP3 to lipid bilayers

(A) Liposome sedimentation assay of fused σ3-δ hemicomplex binding to liposomes enriched in the indicated phosphoinositides. Fractions labelled as (S) supernatant and (P) pellet.

(B) Quantification of fused σ3-δ hemicomplex band intensity in the pelleted fraction across indicated phoshoinositides from the liposome sedimentation assay shown in (A). Liposome sedimentation assays were performed in triplicate (n=3).

(C) Liposome SPR response curve of σ3-δ hemicomplex binding liposomes spiked with 4% PI(3,5)P2 (corresponding to binding curve in Fig 1D), PI(4)P or PI(3)P with PCPE control liposome signal subtracted. Concentrations of σ3-δ hemicomplex annotated by colour key.

(D) Low magnification cryo-EM screening image AP3:ARF1 coated tubules on a grid hole. Scale bar 500 nm.

(E) Computational slice through a cryo-electron tomogram of AP3 coated tubules used for subtomogram averaging. Scale bar 200 nm.

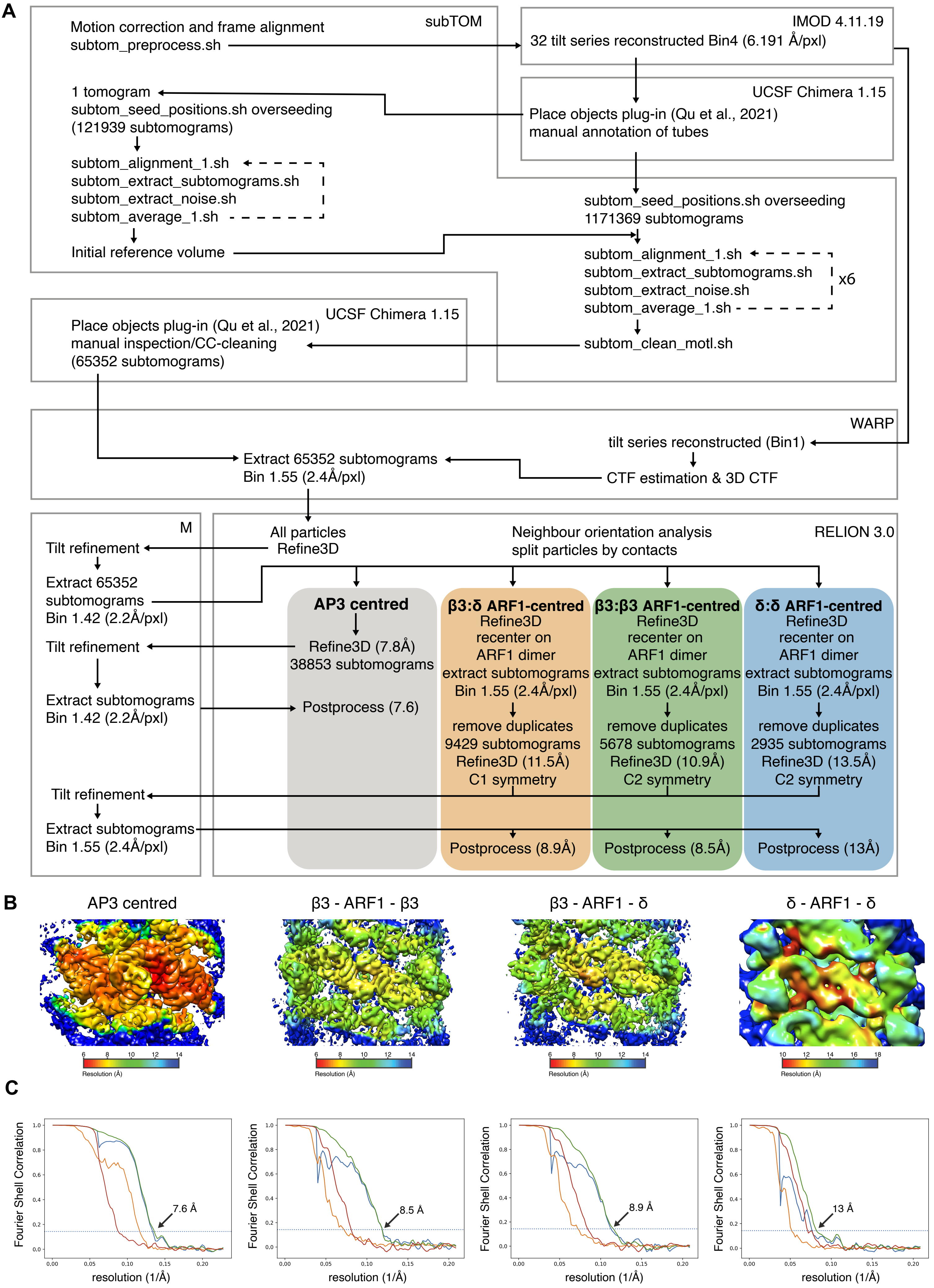
A
subTOM
Motion correction and frame alignment
subtom_preprocess.sh
IMOD 4.11.19
32 tilt series reconstructed Bin4 (6.191 Å/pxl)
UCSF Chimera 1.15
Place objects plug-in (Qu et al., 2021)
manual annotation of tubes
1 tomogram
subtom_seed_positions.sh overseeding
(121939 subtomograms)
subtom_alignment_1.sh
subtom_extract_subtomograms.sh
subtom_extract_noise.sh
subtom_average_1.sh
Initial reference volume
subtom_seed_positions.sh overseeding
1171369 subtomograms
subtom_alignment_1.sh
subtom_extract_subtomograms.sh
subtom_extract_noise.sh
subtom_average_1.sh
x6
subtom_clean_motl.sh
UCSF Chimera 1.15
Place objects plug-in (Qu et al., 2021)
manual inspection/CC-cleaning
(65352 subtomograms)
WARP
tilt series reconstructed (Bin1)
CTF estimation & 3D CTF
Extract 65352 subtomograms
Bin 1.55 (2.4Å/pxl)
M
Tilt refinement
Extract 65352
subtomograms
Bin 1.42 (2.2Å/pxl)
Tilt refinement
Extract subtomograms
Bin 1.42 (2.2Å/pxl)
Tilt refinement
Extract subtomograms
Bin 1.55 (2.4Å/pxl)
RELION 3.0
All particles
Refine3D
Neighbour orientation analysis
split particles by contacts
AP3 centred
Refine3D (7.8Å)
38853 subtomograms
Postprocess (7.6)
β3:δ ARF1-centred
Refine3D
recenter on
ARF1 dimer
extract subtomograms
Bin 1.55 (2.4Å/pxl)
remove duplicates
9429 subtomograms
Refine3D (11.5Å)
C1 symmetry
Postprocess (8.9Å)
β3:β3 ARF1-centred
Refine3D
recenter on
ARF1 dimer
extract subtomograms
Bin 1.55 (2.4Å/pxl)
remove duplicates
5678 subtomograms
Refine3D (10.9Å)
C2 symmetry
Postprocess (8.5Å)
δ:δ ARF1-centred
Refine3D
recenter on
ARF1 dimer
extract subtomograms
Bin 1.55 (2.4Å/pxl)
remove duplicates
2935 subtomograms
Refine3D (13.5Å)
C2 symmetry
Postprocess (13Å)
B
AP3 centred
β3 - ARF1 - β3
β3 - ARF1 - δ
δ - ARF1 - δ
Resolution (Å)
C
Fourier Shell Correlation
resolution (1/Å)
7.6 Å
8.5 Å
8.9 Å
13 Å

Fig. S2. Computational image processing pipeline
(A) Summary of cryo-ET and subtomogram averaging pipeline for AP3-centred (grey) and linkage-centred β3 – ARF1 dimer – δ (orange), β3 – ARF1 dimer – β3 (green), δ – ARF1 dimer – δ (blue) reconstructions.
(B) Isosurface maps coloured by local resolution for the AP3-centred and linkage-centred reconstructions.
(C) Fourier shell correlation plots for the indicated reconstructions. Coloured as: unmasked map (orange), masked map (green), phase randomized masked map (red), corrected FSC (blue).

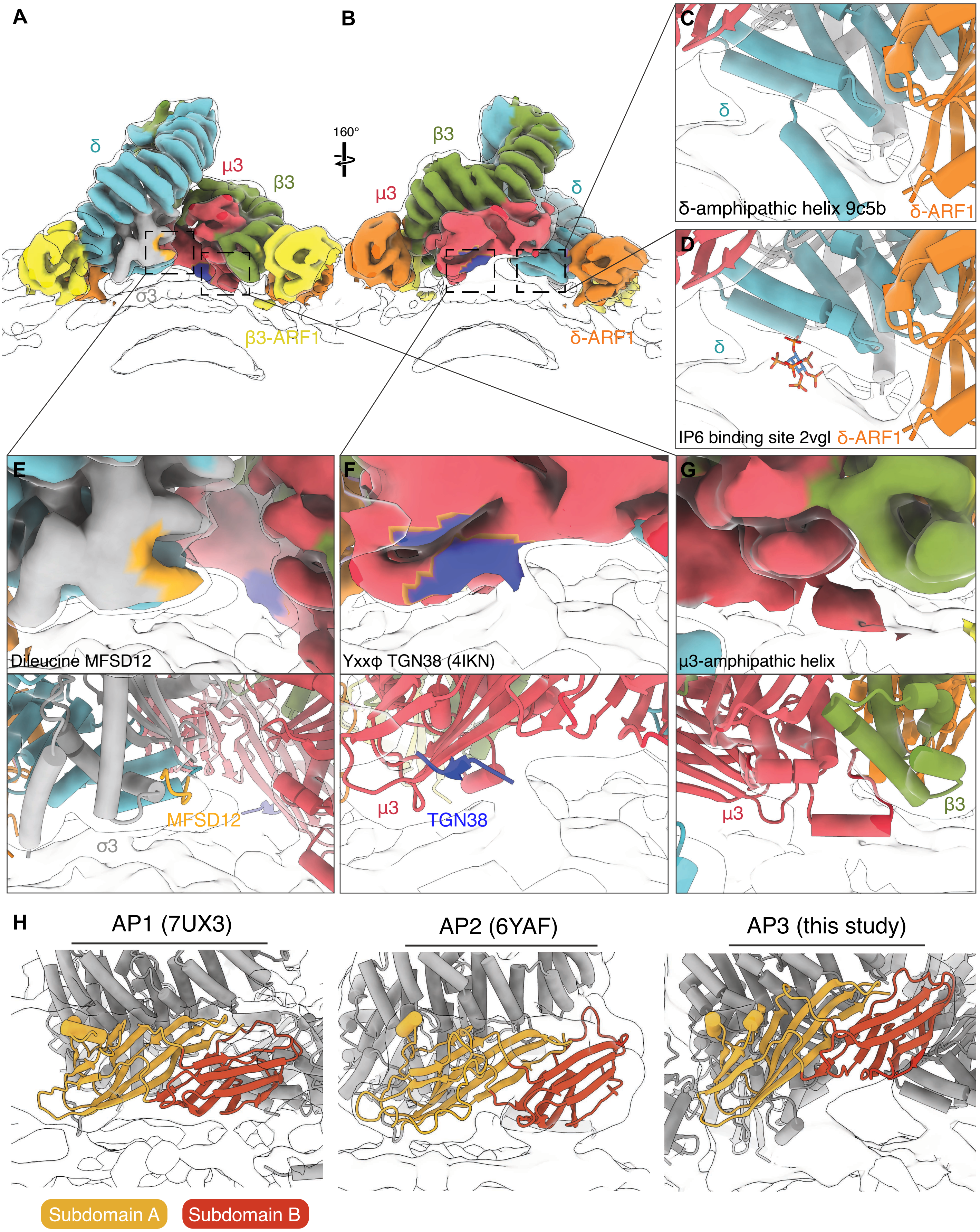

Fig. S3. Interactions between AP3 and the membrane

(A and B) Isosurface view of the AP3-ARF1 reconstruction coloured according to component subunit, and additionally shown as a transparent, low-pass filtered, lower-isosurface threshold reconstruction where the membrane is visible.

Panels depict expanded views of: **(C)** the proposed amphipathic helix at δ N-terminus as modelled in 9C5B(15); **(D)** Inositol hexaphosphate as previously resolved in AP2 α phosphoinositide binding site from crystal structure 2VGL(3), (the binding mode is equivalent in a similar structure of AP2 bound to PI(4,5)P2 (8T1O)(59); **(E)** AlphaFold3 prediction of MFSD12 peptide bound to the previously described dileucine binding site homologous to other AP complexes **(F)** the TGN38 peptide bound to the YxxΦ binding site of μ3 (4IKN)(31); **(G)** the AF3-predicted amphipathic helix within the μ3 linker (μ3-amphipathic helix).

(H) Structural comparison of the interactions of μ domains with the membrane showing C-μ1 (7UX3)(26) and C-μ2 (6YAF)(27) laying flat on the membranes contacting with both subdomains A and B while C-μ3 (this study) only contacts with subdomain A. Subdomains A and B coloured in yellow and red respectively.

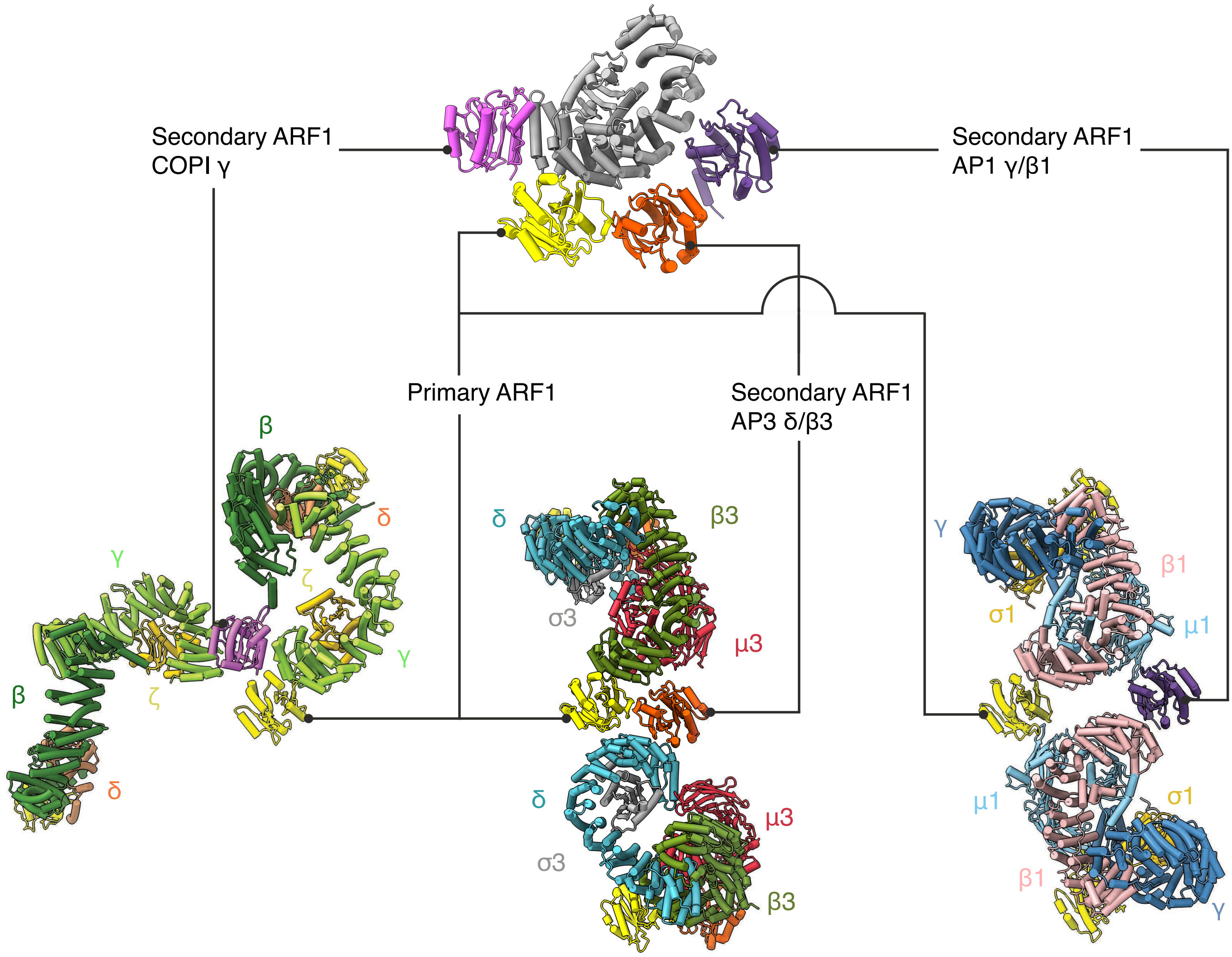

Fig. S4. Comparison of ARF1 binding sites across AP family members
Comparison of primary and distinct secondary ARF1 binding sites that mediate lattice formation between COPI, AP3 and AP1. Depicts how the binding of a secondary ARF1 coordinates the relative angle and position of the next complex to mediate lattice formation and carrier morphology.

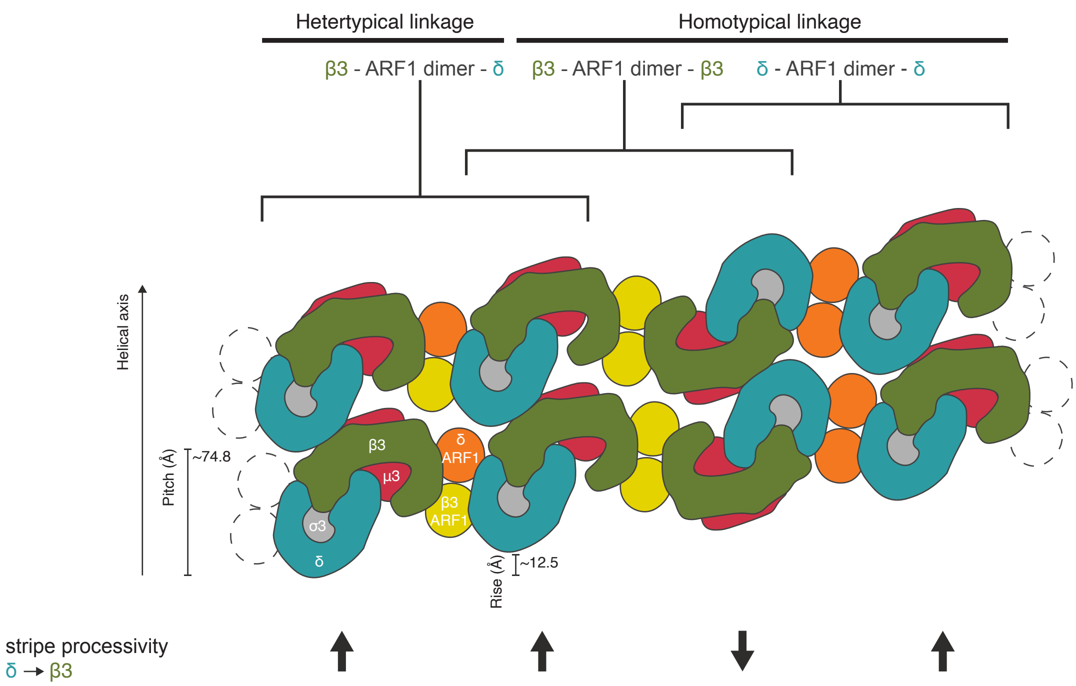

Fig. S5. Schematic diagram of ARF1-dimer mediated linkages

Schematic diagram of heterotypic (δ:β3) and homotypic (δ:δ and β3:β3) linkage types mediated by the ARF1 dimer. Arrows (bold) the direction of an individual AP3 stripe from δ to β3 of an individual AP3 complex. Approximate values for pseudo helical pitch (~74.8 Å) and rise (~12.5 Å) annotated.

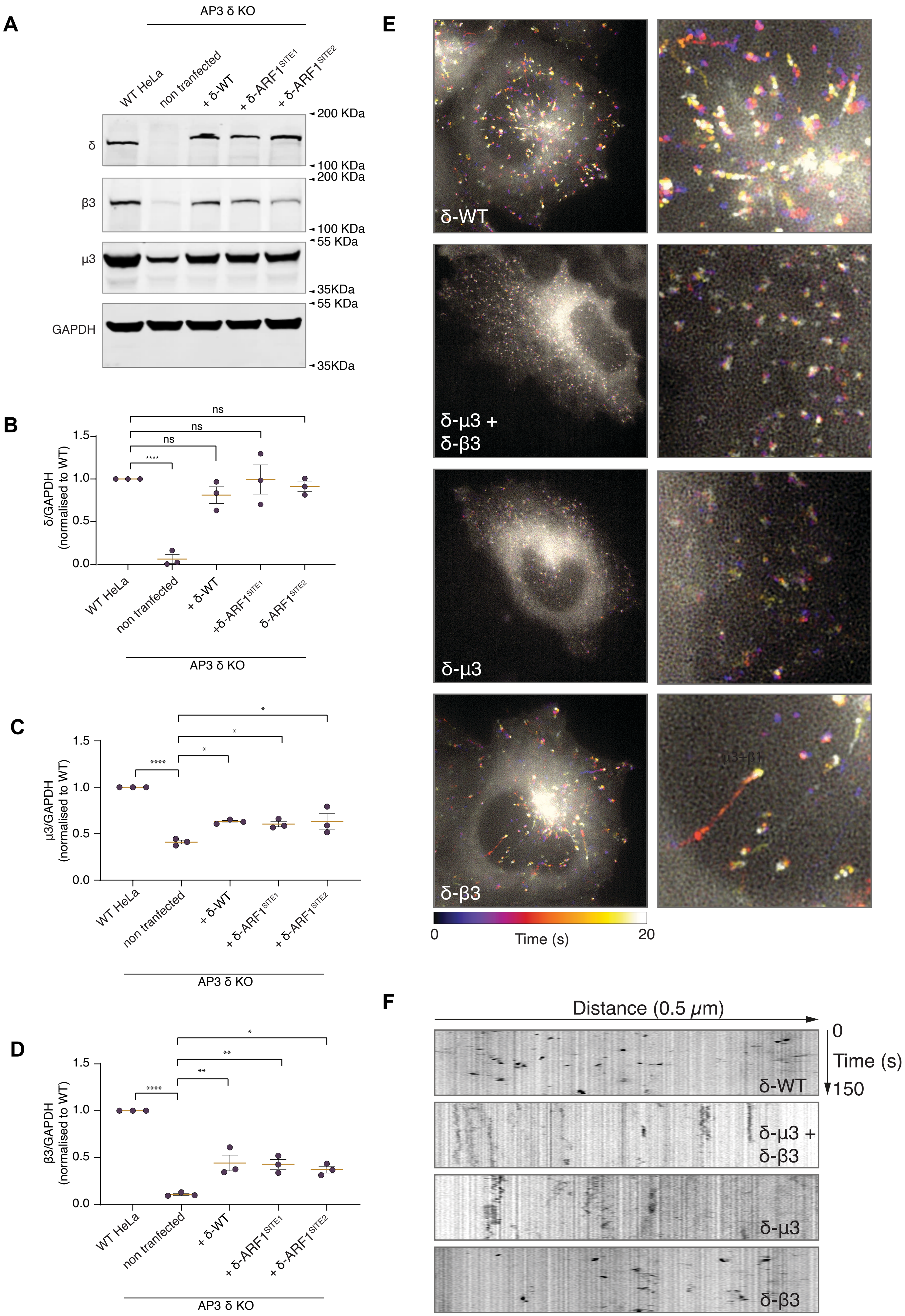

A
AP3 δ KO
WT HeLa
non tranfected
+ δ-WT
+ δ-ARF1SITE1
+ δ-ARF1SITE2
200 KDa
100 KDa
200 KDa
100 KDa
55 KDa
35KDa
55 KDa
35KDa
δ
β3
µ3
GAPDH
B
ns
ns
ns
****
δ/GAPDH (normalised to WT)
1.5
1.0
0.5
0.0
WT HeLa
non tranfected
+ δ-WT
+δ-ARF1SITE1
δ-ARF1SITE2
AP3 δ KO
C
*
*
*
****
µ3/GAPDH (normalised to WT)
1.5
1.0
0.5
0.0
WT HeLa
non tranfected
+ δ-WT
+ δ-ARF1SITE1
+ δ-ARF1SITE2
AP3 δ KO
D
*
**
**
****
β3/GAPDH (normalised to WT)
1.5
1.0
0.5
0.0
WT HeLa
non tranfected
+ δ-WT
+ δ-ARF1SITE1
+ δ-ARF1SITE2
AP3 δ KO
E
δ-WT
δ-µ3 +
δ-β3
δ-µ3
δ-β3
0
Time (s)
20
F
Distance (0.5 µm)
0
Time (s)
150
δ-WT
δ-µ3 +
δ-β3
δ-µ3
δ-β3

Fig. S6. AP-3 δ subunit KO destabilises the AP3 complex and interface-rescue mutations restore complex integrity but not carrier motility

(A) Western Blots from a single representative experiment showing the relative abundance of δ, β3 and μ3 in WT HeLa Cas9 cells, AP3D1 KO HeLa Cas9 cells and AP3D1 KO HeLa Cas9 cells expressing δ-WT-SG, δ-ARF1Site1-SG (δF77S, δM110S, δL111S) and δ-ARF1Site2-SG (δH157D, δK159D, δR187D, δR163D). GAPDH was used as a loading control. Quantification of the change in abundance of **(B)** δ **(C)** β3 and **(D)** μ3 normalised to GAPDH and relative to the ratio obtained from WT HeLa Cas9 cells. Statistical analysis = One-Way ANOVA and Tukey Post-Hoc Test. Mean ± SEM of three experiments. *, $p < 0.05$; **, $p < 0.01$, ****, $p < 0.001$.

(E) Time colour coded max projection of δ-WT-SG positive dots (represented by the RGB colour bar 0s-20s) shows their trajectory over time (shown as a max projection, generated from Fig. 4C on 20 frames total). The insert shows a close-up example.

(F) Kymographs showing the movement of δ-WT-SG structures corresponding to Fig. 4C and movie S2.

Sample	AP3 tubules			
**Data acquisition**				
Microscope	FEI Titan Krios (G4)			
Voltage (keV)	300			
Energy-filter (eV)	20			
Detector	Falcon 4i			
Pixel size (Å)	1.548			
Defocus range (microns)	-2.5 to -6.0			
Acquisition scheme	-60/+60˚, 3˚			
Total dose (electrons/Å^2)	~120 (3 per tilt)			
Dose rate (electrons/Å^2/sec)	~2.66			
EER frames	360			
Tomogram number	32			
**Image processing**	**AP3-centered**<br>PDB 9RTW<br>EMDB-54255	**β3-ARF1-β3 ARF1-centered**<br>PDB 9RTX<br>EMDB-54256	**β3-ARF1-δ ARF1-centered**<br>PDB 9RTY<br>EMDB-54257	**δ-ARF1-δ ARF1-centered**<br>PDB 9RTZ<br>EMDB-54258
Subtomograms	38853	5678	9429	2935
Symmetry	C1	C2	C1	C2
Resolution at 0.143 FSC (Å)	7.6	8.5	8.9	13
B-factor	-100.000	-288.542	-585.741	-151.302

Table S1. Cryo-ET data acquisition and image processing parameters

Target name	Sequence
AP3D1_1_F	GAACTCCCCGCAGATCCAGG
AP3D1_2_F	ACTTGCGGATGGCCTTCACG
AP3B1_1_F	GAGCATATCGAGTTAGCATG
AP3B1_2_F	GGAATTATCTAATTGGCCAG

Table S2. CRISPR RNA guides

Movie S1. Mutagenesis of ARF1 binding sites ablates AP3 recruitment in cells
Lattice-SIM live-cell imaging of a HeLa cell stably expressing AP3D1-WT-SG, AP3D1-Site1+Site2-SG (δF77S, δM110S, δL111S+δH157D, δK159D, δR187D, δR163D), AP3D1-Site1-SG (δF77S, δM110S, δL111S) or AP3D1-Site2-SG (δH157D, δK159D, δR187D, δR163D). Endogenous AP3D1 was abolished using transient CRISPR-KO. All cells were imaged every 1.39s for 2.29 min. Scale bar: 10 μm

Movie S2. Mutagenesis of inter-AP3 contacts disrupts carrier motility and morphology
Lattice-SIM live-cell imaging of a HeLa cell stably expressing AP3D1-WT-SG, AP3D1-(δ-μ3 + δ-β3)-SG (E313K, D314K, D316K + K342D, K371D, K378D, K379D, K386D), AP3D1-(δ-μ3)-SG (E313K, D314K, D316K) or AP3D1-(δ-β3)-SG (K342D, K371D, K378D, K379D, K386D). Endogenous AP3D1 was abolished using transient CRISPR-KO. All cells were imaged every 1.39s for 2.29 min. Scale bar: 10 μm
